# Estimated small dense low-density lipoprotein cholesterol and hyperuricemia in diabetic patients

**DOI:** 10.3389/fendo.2026.1790201

**Published:** 2026-03-17

**Authors:** Mengjiao Xu, Han Yan, Yi Xue, Yong Yin, Xuejing Shao, Qichao Yang

**Affiliations:** Department of Endocrinology, Wujin Clinical College of Xuzhou Medical University, Affiliated Wujin Hospital of Jiangsu University, Changzhou, Jiangsu, China

**Keywords:** diabetes, hyperuricemia, lipids, low-density lipoprotein cholesterol, nonlinear relationship

## Abstract

**Introduction:**

Small dense low-density lipoprotein cholesterol (sdLDL-C) is a key driver of atherosclerotic cardiovascular disease risk. This study aims to investigate the relationship between estimated sdLDL-C (E-sdLDL-C) and hyperuricemia in diabetic populations.

**Methods:**

This study analyzed 3572 diabetic participants from the NHANES dataset and an independent validation cohort of 248 Chinese subjects from the Affiliated Wujin Hospital of Jiangsu University. E-sdLDL-C was derived from basic lipid profile parameters. Hyperuricemia was determined by serum uric acid ≥420 μmol/L for men and ≥360 μmol/L for women. The relationship between E-sdLDL-C and hyperuricemia was examined using logistic regression, with restricted cubic splines applied to explore non-linear associations.

**Results:**

Diabetic patients with hyperuricemia had significantly higher E-sdLDL-C levels (P<0.001). Each standard deviation increase in E-sdLDL-C was associated with 39% higher odds of hyperuricemia (OR = 1.39, 95% CI: 1.28-1.51, P<0.001). Quartile analysis showed a dose-response relationship, with progressively higher odds ratios across increasing quartiles of E-sdLDL-C. Restricted cubic spline modeling identified a non-linear relationship, with an inflection point at 25.83 μmol/L. The robustness of these associations was confirmed through external validation in an independent Chinese diabetic cohort.

**Conclusion:**

E-sdLDL-C might serve as a practical biomarker for identifying diabetic patients at increased hyperuricemia risk.

## 1 Introduction

Hyperuricemia, defined by persistently elevated serum uric acid (SUA) concentrations, constitutes a metabolic disorder that significantly increases susceptibility to gout, renal disease, hypertension, and cardiovascular conditions (1–5). Consequently, effective SUA control has emerged as an important therapeutic objective for improving prognosis and preventing associated complications.

Elevated low-density lipoprotein (LDL) particle numbers represent a key determinant of atherosclerotic cardiovascular disease (ASCVD) risk (6, 7). LDL particles display substantial physicochemical diversity in terms of size, density, electrical charge, and molecular composition, factors that modulate their atherogenic properties (8). Compared to large buoyant LDL (lbLDL), small dense LDL (sdLDL) demonstrates enhanced atherogenicity, characterized by extended plasma residence time, diminished LDL receptor binding affinity, superior arterial intimal penetration capacity, and heightened oxidative vulnerability (8). Numerous studies suggest that sdLDL-C, which characterizes LDL particle subfractions, serves as a more significant independent determinant of ASCVD risk (9). However, direct sdLDL quantification requires sophisticated methodologies with significant technical and economic barriers, restricting widespread clinical implementation (10). To overcome this constraint, Sampson et al. and colleagues formulated an estimation equation (estimated sdLDL-C; E-sdLDL-C) derived from conventional lipid parameters, offering a practical clinical assessment tool (11). In the UK Biobank primary prevention cohort, E-sdLDL-C exhibited superior performance in predicting overall ASCVD risk relative to established biomarkers including LDL-C and apolipoprotein B (12).

Diabetes and hyperuricemia demonstrate frequent comorbidity (13, 14). Epidemiological investigations reveal markedly elevated hyperuricemia risk among diabetic populations compared to non-diabetic individuals (14, 15). The coexistence of diabetes and hyperuricemia not only amplifies risks of renal damage and urinary calculus formation but may also intensify cardiovascular morbidity through multiple pathophysiological pathways encompassing augmented oxidative stress, elevated inflammatory cytokine production, impaired endothelial function, and aggravated insulin resistance (16). Within this framework, E-sdLDL-C, as a novel cardiovascular risk indicator, holds potential utility for identifying high-risk cardiometabolic phenotypes among patients with concurrent diabetes and hyperuricemia. Although prior research documents positive correlations between sdLDL-C concentrations and SUA levels, most studies have concentrated on general community or non-diabetic populations, with diabetic cohort evidence remaining sparse (17, 18). The present investigation consequently evaluates the relationship between E-sdLDL-C and hyperuricemia in diabetic individuals.

## 2 Materials and methods

### 2.1 Study cohort

**Figure 1:**
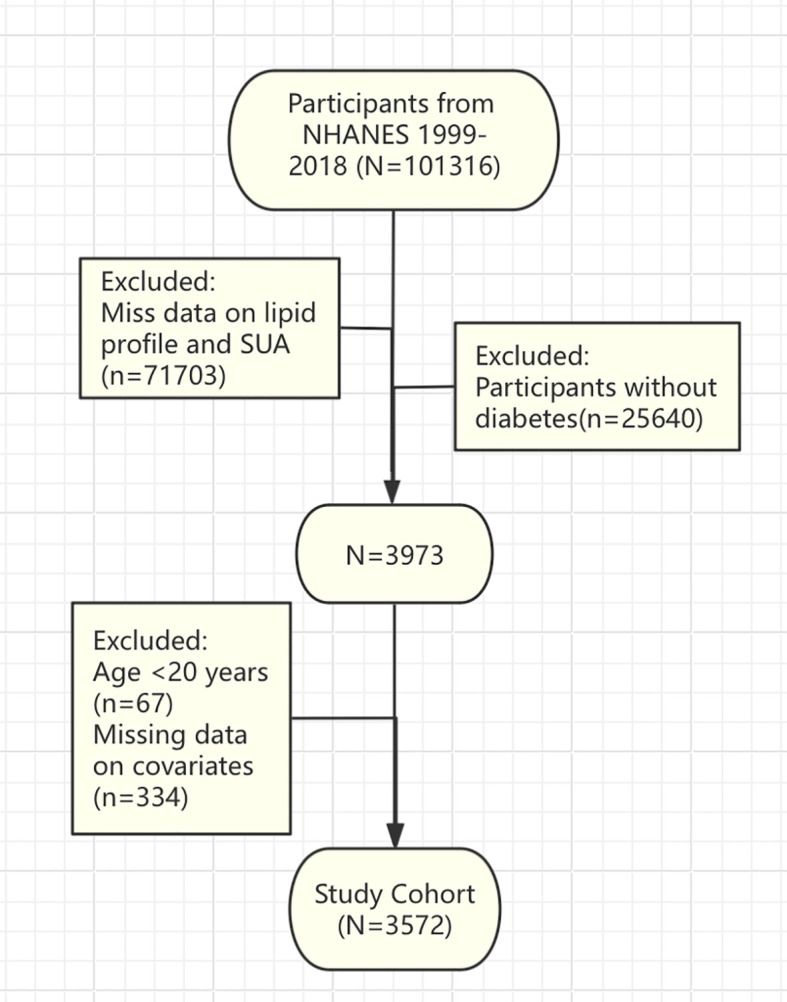
Flowchart illustrating participant selection process from NHANES.

The present study employed publicly accessible data derived from the National Health and Nutrition Examination Survey (NHANES), a nationally representative surveillance program implementing a stratified, multistage probability sampling methodology to systematically acquire demographic characteristics, lifestyle factors, laboratory parameters, and health outcome data from the United States population. Data from ten consecutive NHANES survey cycles conducted between 1999 and 2018 were pooled, yielding an initial sample of 101316 participants. Exclusion criteria encompassed individuals younger than 20 years, participants without diabetes diagnosis, and those lacking complete data regarding lipid profiles, SUA measurements, or other relevant covariates. Diabetes status was determined according to any of the following criteria: self-reported physician diagnosis, fasting plasma glucose (FPG) ≥7.0 mmol/L, hemoglobin A1c (HbA1c) ≥6.5%, or current pharmacological treatment for diabetes. Application of these selection criteria resulted in a final analytical sample comprising 3572 diabetic participants (**Figure 1**).

For external validation purposes, diabetes patients attending health education programs at the Department of Endocrinology, Affiliated Wujin Hospital of Jiangsu University, were enrolled between January 2024 and January 2025. Inclusion criteria comprised: (1) age ≥20 years, (2) confirmed diabetes diagnosis according to American Diabetes Association standards, and (3) availability of complete lipid profile and SUA measurements. Exclusion criteria included: (1) severe renal impairment, (2) active malignancy, (3) pregnancy or lactation, and (4) use of medications known to significantly affect uric acid metabolism. The validation cohort consisted of 248 individuals (132 males, 116 females) with mean age 62.93 years. The study protocol obtained ethical approval from the Institutional Review Board of Affiliated Wujin Hospital of Jiangsu University (2026-SR-020), with all participants providing written informed consent prior to enrollment.

### 2.2 Exposure and outcome

E-sdLDL-C levels were calculated using a newly derived model derived from the standard lipid panel, as introduced by the Sampson equation (ElbLDL-C = 1.43 × LDL-C - (0.14 × (ln(triglycerides[TG]) × LDL-C)) - 8.99; E-sdLDL-C = LDL-C − ElbLDL-C) (11, 12). This estimation method was selected because direct measurement of sdLDL-C is costly and technically demanding, whereas the Sampson formula has been validated against direct assays and independently predicts atherosclerotic cardiovascular disease risk, making it suitable for large-scale epidemiological studies. On the other hand, the diagnostic threshold for hyperuricemia is established at SUA levels ≥420 µmol/L for males and ≥360 µmol/L for females (19).

### 2.3 Covariates

Multivariable analyses incorporated adjustment for an extensive array of potential confounding variables, selected based on established literature and clinical relevance to hyperuricemia in diabetic populations (20–22), spanning several domains: (1) demographic characteristics, comprising age, sex, race/ethnicity (categorized as Mexican American, non-Hispanic Black, non-Hispanic White, other Hispanic, and other races), educational attainment (above high school or not), and marital status (married or not); (2) lifestyle factors, specifically smoking history and alcohol use (defined as ≥12 alcoholic beverages annually); (3) anthropometric and clinical parameters, including body mass index (BMI) and comorbid conditions such as hypertension and cardiovascular diseases (CVDs). Hypertension was ascertained based on self-reported physician diagnosis, while CVDs encompassed self-reported history of myocardial infarction, stroke, heart failure, coronary heart disease, or angina pectoris; and (4) laboratory indices, including hemoglobin A1c (HbA1c), albumin, TG, total cholesterol (TC), LDL-C, high-density lipoprotein cholesterol (HDL-C), and serum creatinine (Scr).

The external validation cohort derived from Affiliated Wujin Hospital of Jiangsu University provided analogous data, specifically including demographic variables (age, sex, smoking status, alcohol use), medical history (hypertension and CVDs), BMI, and fasting laboratory measurements encompassing albumin, HbA1c, TG, TC, LDL-C, HDL-C, and Scr.

### 2.4 Statistical analysis

Analyses employed the designated weighting scheme. Continuous variables are presented as weighted means with 95% confidence intervals (CI); categorical variables as unweighted counts and weighted percentages. Group comparisons used t-tests for continuous variables and chi-square tests for categorical variables. Associations between E-sdLDL-C and hyperuricemia/SUA were examined using weighted logistic/linear regression models: Model 1 (unadjusted), Model 2 (adjusted for age, sex, race, education, marital status, smoking, and alcohol use), and Model 3 (further adjusted for hypertension, CVDs, BMI, albumin, Hba1c, and Scr). SHapley Additive exPlanations (SHAP) analysis elucidated model behavior and covariate contributions. Non-linear relationships were assessed using four-knot restricted cubic splines with median E-sdLDL-C as reference. The optimal E-sdLDL-C threshold was determined by maximizing model likelihood across candidate cut points. A piecewise logistic regression model was then applied to estimate associations with hyperuricemia risk above and below this threshold. Stratified analyses evaluated effect modification by covariates. External validation utilized an independent diabetes cohort from the Affiliated Wujin Hospital of Jiangsu University. All analyses were performed in R with statistical significance defined as two-sided P<0.05.

## 3 Results

### 3.1 Baseline characteristics

(**Table 1**) presents baseline characteristics of diabetic participants stratified by hyperuricemia status. In terms of demographic and sociodemographic characteristics, participants with hyperuricemia were older and more likely to be female (P<0.001). The ethnic distribution differed significantly between groups, whereas marital status and educational attainment were comparable (P>0.05). Lifestyle factors, including smoking and alcohol use, also did not differ significantly (P>0.05). Regarding clinical characteristics, the hyperuricemia group had a higher BMI and a greater prevalence of hypertension and CVDs (P<0.001). HbA1c levels were also higher among hyperuricemic participants (P<0.001). Biochemically, hyperuricemic participants exhibited a more atherogenic lipid profile (higher TG, lower HDL-C, and elevated E-sdLDL-C) and higher Scr levels (P<0.05), whereas TC and LDL-C did not differ significantly between groups.

**Table 1:** Baseline characteristics of diabetic participants by hyperuricemia status.

Variables	Overall (n=3572)	Non-hyperuricemia (n=2552)	Hyperuricemia (n=1020)	P value
Age (years)	59.16 (58.52, 59.81)	58.13 (57.42, 58.84)	61.77 (60.64, 62.90)	<0.001
Sex, n (%)				<0.001
Female	1691 (48.04%)	1145 (45.35%)	546 (54.81%)	
Male	1881 (51.96%)	1407 (54.65%)	474 (45.19%)	
Race, n (%)				<0.001
Mexican American	688 (8.88%)	571 (10.54%)	117 (4.70%)	
Other Hispanic	348 (5.80%)	272 (6.57%)	76 (3.86%)	
Non-Hispanic White	1374 (64.28%)	937 (63.12%)	437 (67.22%)	
Non-Hispanic Black	857 (13.66%)	552 (12.45%)	305 (16.73%)	
Other Race	305 (7.37%)	220 (7.31%)	85 (7.50%)	
Married, n (%)	2006 (59.47%)	1457 (60.12%)	549 (57.84%)	0.385
Above high school, n (%)	1472 (49.71%)	1027 (48.63%)	445 (52.43%)	0.107
Smokers, n (%)	1817 (51.05%)	1302 (51.12%)	515 (50.88%)	0.934
Alcohol use, n (%)	2401 (70.58%)	1728 (71.34%)	673 (68.65%)	0.225
Hypertension, n (%)	2294 (63.31%)	1504 (57.82%)	790 (77.15%)	<0.001
CVDs, n (%)	902 (23.86%)	573 (21.77%)	329 (29.12%)	<0.001
BMI (kg/m2)	32.98 (32.61, 33.35)	32.02 (31.57, 32.47)	35.42 (34.67, 36.17)	<0.001
Albumin (g/L)	41.08 (40.90, 41.26)	41.22 (41.01, 41.42)	40.74 (40.40, 41.08)	0.020
HbA1c (%)	7.03 (6.96, 7.10)	6.79 (6.70, 6.89)	7.12 (7.04, 7.21)	<0.001
TG (mg/dL)	151.62 (146.97, 156.26)	146.33 (141.44, 151.22)	164.95 (156.36, 173.55)	<0.001
TC (mg/dL)	183.91 (181.57, 186.25)	183.45 (180.78, 186.12)	185.07 (181.11, 189.04)	0.480
HDL-C (mg/dL)	48.95 (48.19, 49.72)	49.60 (48.70, 50.49)	47.33 (46.26, 48.40)	0.001
LDL-C (mg/dL)	105.36 (103.48, 107.23)	105.22 (103.20, 107.25)	105.69 (102.43, 108.95)	0.794
E-sdLDL-C (mg/dL)	36.35 (35.63, 37.07)	35.77 (34.97, 36.58)	37.79 (36.55, 39.03)	0.004
Scr (umol/L)	84.60 (82.30, 86.90)	80.05 (77.46, 82.63)	96.08 (92.81, 99.34)	<0.001
SUA (μmol/L)	346.55 (342.42, 350.68)	305.19 (301.87, 308.52)	450.90 (445.28, 456.51)	<0.001

Continuous variables: weighted means (95% CI); categorical variables: unweighted counts (weighted %).

### 3.2 Regression analysis of the relationship between E-sdLDL-C and hyperuricemia

**Figure 2:**
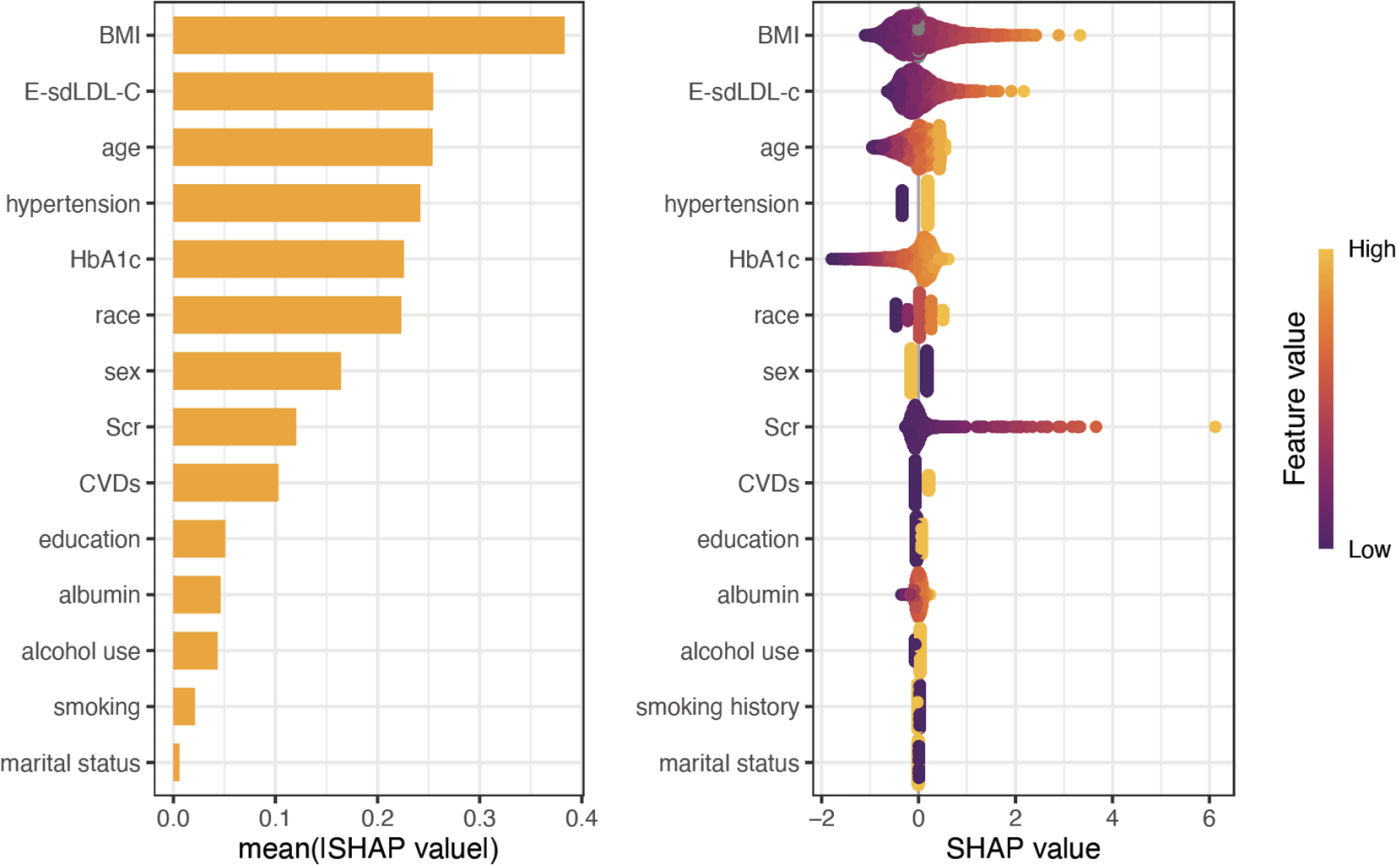
Feature importance assessment via SHAP analysis.

(**Table 2**) summarizes logistic/linear regression analyses assessing the relationship between E-sdLDL-C and both hyperuricemia and SUA concentrations. After full adjustment (Model 3), each standard deviation increment in E-sdLDL-C corresponded to a 39% elevated hyperuricemia risk (OR = 1.39, 95% CI: 1.28-1.51, P<0.001). Quartile-based analysis revealed a graded association: compared to the lowest quartile (Q1), participants in Q4 had 2.29 times higher odds of hyperuricemia (OR = 2.29, 95% CI: 1.80-2.91, P<0.001), with significant trend across quartiles (P for trend <0.001). Similarly, each standard deviation increase in E-sdLDL-C was linked to a 14.79 μmol/L rise in SUA levels (β=14.79, 95% CI: 11.96-17.62, P<0.001), while Q4 participants exhibited 35.72 μmol/L higher SUA than Q1 (β=35.72, 95% CI: 27.68-43.75, P<0.001). On the other hand, feature importance was quantified using SHAP analysis to interpret the hyperuricemia risk assessment model in diabetic patients (**Figure 2**). The mean absolute SHAP values ranked covariates by their contribution magnitude, revealing a hierarchical structure of predictor importance. E-sdLDL-C emerged as the second most influential factor associated with hyperuricemia risk in the diabetic cohort, following BMI. Directional effects assessed via SHAP summary plots demonstrated consistent positive associations between hyperuricemia risk and elevated E-sdLDL-C levels.

**Table 2:** Regression analyses of E-sdLDL-C associations with hyperuricemia and SUA.

E-sdLDL-C	Model 1	Model 2	Model 3
Hyperuricemia	OR (95%CI) P value		
Per SD increase	1.16 (1.08, 1.25) <0.001	1.27 (1.18, 1.37) <0.001	1.39 (1.28, 1.51) <0.001
Quantiles			
Q1	Reference	Reference	Reference
Q2	1.42 (1.15, 1.75) 0.001	1.51 (1.22, 1.88) <0.001	1.49 (1.18, 1.87) <0.001
Q3	1.30 (1.05, 1.61) 0.016	1.50 (1.20, 1.86) <0.001	1.64 (1.30, 2.06) <0.001
Q4	1.48 (1.20, 1.83) <0.001	1.90 (1.52, 2.37) <0.001	2.29 (1.80, 2.91) <0.001
P for tend	0.001	<0.001	<0.001
SUA levels	β (95%CI) P value		
Per SD increase	6.26 (3.23, 9.29) <0.001	11.89 (8.91, 14.86) <0.001	14.79 (11.96, 17.62) <0.001
Quantiles			
Q1	Reference	Reference	Reference
Q2	11.93 (3.36, 20.50) 0.006	18.07 (9.84, 26.30) <0.001	15.25 (7.48, 23.02) <0.001
Q3	12.01 (3.45, 20.58) 0.006	22.32 (14.03, 30.60) <0.001	23.64 (15.83, 31.45) <0.001
Q4	13.99 (5.42, 22.55) 0.001	30.15 (21.72, 38.58) <0.001	35.72 (27.68, 43.75) <0.001
P for tend	0.002	<0.001	<0.001

OR: odds ratio. 95% CI: 95% confidence interval. Model 1: non-adjusted. Model 2: adjusted for age, sex, race, education, marital status, smoking, and alcohol use. Model 3: adjusted for Model 2+hypertension, CVDs, BMI, albumin, Hba1c, and Scr.

### 3.3 Nonlinear and subgroup analyses

**Figure 3:**
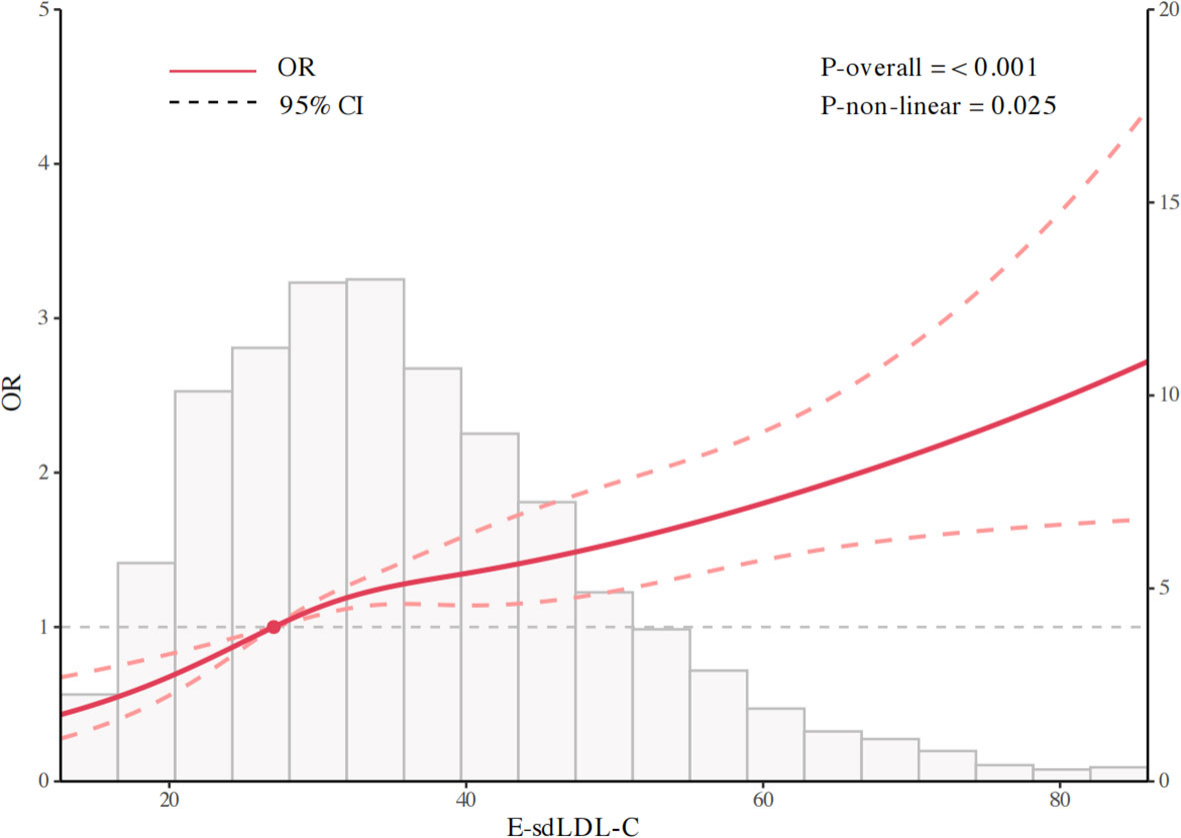
Restricted cubic spline analysis depicting non-linear relationship.

**Figure 4:**
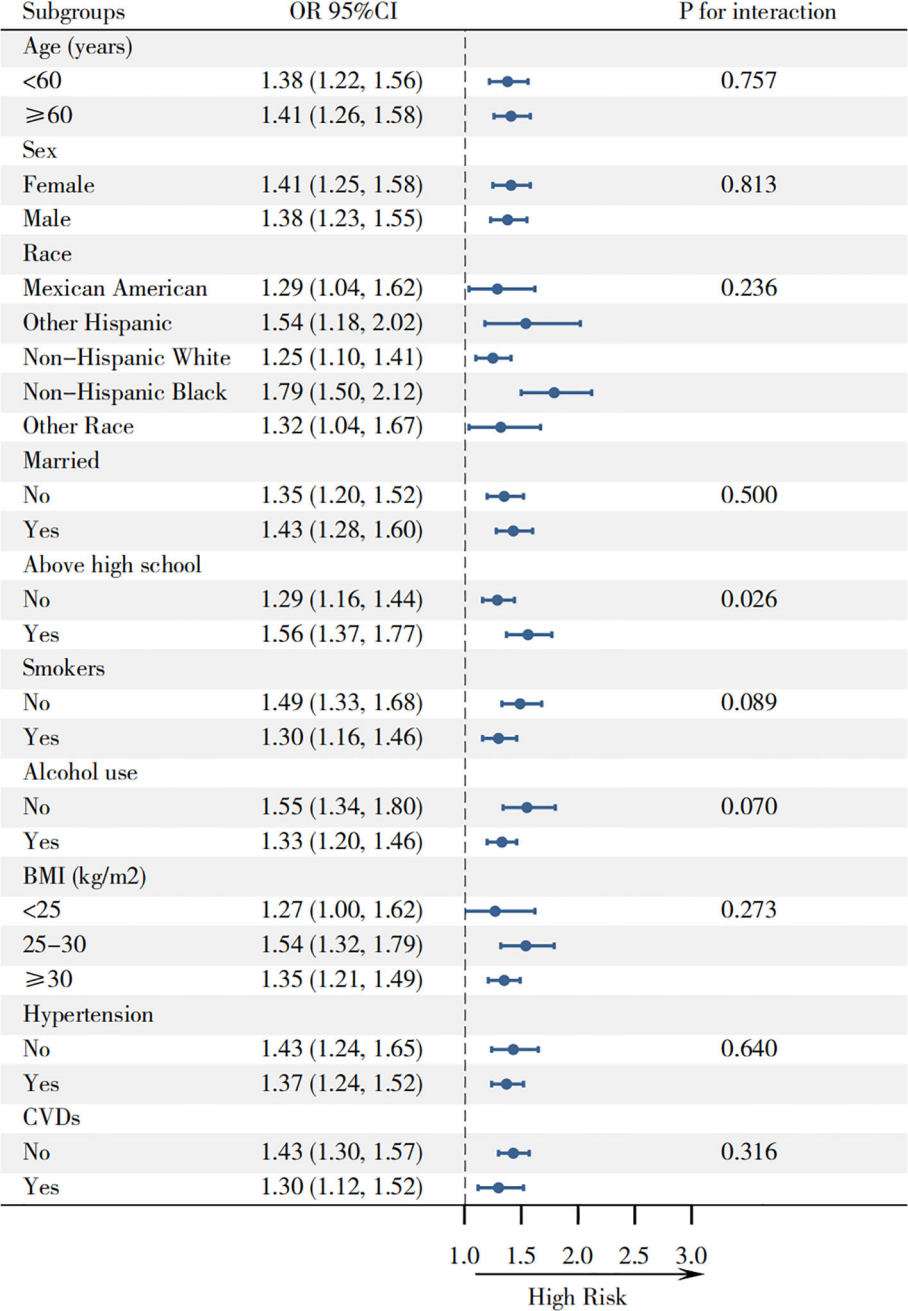
Forest plot of subgroup analyses examining effect modification.

Using restricted cubic spline modeling, we identified a significant non-linear positive correlation between E-sdLDL-C and hyperuricemia risk in patients with diabetes mellitus (overall P<0.001, non-linear P = 0.025) (**Figure 3**). Piecewise regression analysis determined an inflection point at 25.83 μmol/L (**Table 3**). Below this concentration, each standard deviation elevation in E-sdLDL-C was associated with a substantially heightened risk of hyperuricemia (OR = 3.12, 95% CI: 1.81-5.39, P<0.001). Beyond this inflection, the association remained statistically significant but was attenuated (OR = 1.29, 95% CI: 1.17-1.42, P<0.001). Across all evaluated subgroups, heightened E-sdLDL-C concentrations maintained significant associations with hyperuricemia risk (**Figure 4**). Particularly robust correlations emerged in individuals with education above high school (OR = 1.56, 95% CI: 1.37-1.77). The subgroup analysis revealed no evidence of significant effect modification by age, sex, race, marital status, smoking status, alcohol use, BMI categories (<25/25-30/≥30 kg/m^2^), hypertension, or CVDs (all P for interaction >0.05).

**Table 3:** Piecewise regression analysis for E-sdLDL-C threshold identification.

Per SD increase in E-sdLDL-C	OR (95% CI) P value
Hyperuricemia	1.39 (1.28, 1.51) <0.001
Fitting by two-piecewise model	
Inflection point	25.83
E-sdLDL-C < 25.83	3.12 (1.81, 5.39) <0.001
E-sdLDL-C ≥ 25.83	1.29 (1.17, 1.42) <0.001
P for Log-likelihood ratio	0.002

adjusted for age, sex, race, education, marital status, smoking, alcohol use, hypertension, CVDs, BMI, albumin, Hba1c, and Scr.

### 3.4 Validation of external dataset

To validate our findings, we examined the relationship between E-sdLDL-C and hyperuricemia in an independent Chinese diabetic cohort (**Supplementary Material**). Hyperuricemic participants demonstrated significantly higher E-sdLDL-C concentrations relative to their non-hyperuricemic counterparts (**Supplementary Material**, **Supplementary Table 1**) (P<0.001). Logistic regression analyses revealed progressively increasing ORs for hyperuricemia across three adjustment models: unadjusted (OR = 1.79, 95% CI: 1.30-2.45), adjusted for age, sex, smoking status, and alcohol use (OR = 1.90, 95% CI: 1.33-2.71), and further adjusted for hypertension, CVDs, BMI, HbA1c, albumin, and Scr (OR = 1.98, 95% CI: 1.35-2.92) (**Supplementary Material**, **Supplementary Table 2**). Correspondingly, linear regression models demonstrated consistent positive associations between E-sdLDL-C and SUA levels across all adjustment models (β=23.44, 95% CI: 13.13-33.75, P<0.001) (**Supplementary Material**, **Supplementary Table 2**).

## 4 Discussion

This study constitutes a thorough examination of the relationship between E-sdLDL-C and hyperuricemia in diabetic populations, employing both nationally representative survey data and an independent validation cohort. Our results reveal a strong positive association between elevated E-sdLDL-C concentrations and heightened hyperuricemia risk among diabetic individuals, with each standard deviation increase in E-sdLDL-C associated with a 39% elevated risk of hyperuricemia following comprehensive adjustment for covariates.

Clinically, the E-sdLDL-C equation, derived from routine lipid parameters, represents a feasible approach for assessing cardiometabolic risk in diabetic patients. Given that quantification of sdLDL-C necessitates sophisticated methodologies with significant technical and financial constraints, E-sdLDL-C offers a readily available surrogate for stratification of clinical risk (23). Diabetic individuals presenting with both elevated E-sdLDL-C and hyperuricemia likely represent a unique high-risk phenotype requiring intensified therapeutic approaches. Several interconnected mechanistic pathways may account for the association between increased E-sdLDL-C and hyperuricemia in diabetes. Foremost among these is the coexistence of common metabolic abnormalities, including insulin resistance, chronic subclinical inflammation, and enhanced oxidative stress. Insulin resistance promotes hepatic overproduction of uric acid by enhancing purine nucleotide turnover and reducing renal uric acid excretion, leading to hyperuricemia (24, 25). Hyperuricemia further induces endothelial insulin resistance by impairing insulin-stimulated endothelial nitric oxide (NO) synthesis, thereby exacerbating endothelial dysfunction (26, 27). Insulin resistance also stimulates the hepatic synthesis of triglyceride-rich very low-density lipoproteins (VLDL) (28–30). These VLDL particles are subsequently hydrolyzed to generate sdLDL particles (28–30). Due to their specific structural features-such as reduced antioxidant content and altered core lipid composition-as well as prolonged plasma residence time, sdLDL particles are more susceptible to oxidative modification than other LDL subfractions (31, 32). The oxidized form of sdLDL (oxLDL) directly contributes to atherosclerosis by facilitating cholesterol deposition within the subendothelial space and by triggering inflammatory responses and oxidative stress cascades (33, 34). The pro-oxidative environment induced by oxLDL can upregulate xanthine oxidase (XO) expression, which, during uric acid metabolism, generates reactive oxygen species (ROS), thus establishing a positive feedback loop (35–37). Consequently, elevated E-sdLDL-C may reflect not only a more atherogenic lipoprotein profile but also a pro-inflammatory cardiometabolic phenotype that predisposes diabetic individuals to hyperuricemia and its downstream vascular and renal complications. Importantly, SUA abnormalities, including both hyperuricemia and hypouricemia, have also demonstrated clinical relevance in acute care settings, particularly for stroke risk assessment in emergency departments (38, 39). Notably, our analysis identified a nonlinear relationship with an inflection point at 25.83 μmol/L. This threshold may have clinical relevance for risk stratification in diabetic populations and could serve as a reference for clinical risk stratification, although prospective studies are needed for validation.

Several limitations should be noted. First, our findings demonstrate an association rather than causation, and longitudinal studies are needed to establish temporal relationships and potential causal pathways. Importantly, reverse causation (i.e., hyperuricemia influencing lipid metabolism) cannot be excluded. Second, we utilized complete-case analysis by excluding participants with missing data, which may introduce selection bias if the data are not missing completely at random. Additionally, although we adjusted for multiple confounders, residual confounding may persist due to unmeasured factors such as specific medication use (urate-lowering, lipid-lowering, and diuretic agents), dietary information, metabolic comorbidities, and the lack of diabetes type classification data in the NHANES database. Finally, differences in study populations (the NHANES multi-ethnic cohort versus single-center Chinese diabetic patients) and limitations in sample size necessitate future multicenter studies with larger, more diverse populations for robust validation.

## 5 Conclusion

E-sdLDL-C is significantly associated with increased hyperuricemia risk in diabetic populations, suggesting its potential as a practical biomarker for assessing cardiometabolic risk.

## Data Availability

Publicly available datasets were analyzed in this study. This data can be found here: The study utilized data from the publicly accessible NHANES database (https://wwwn.cdc.gov/nchs/nhanes); external validation cohort data can be requested from the corresponding author.
