# Supplementary material for: Estimated small dense low-density lipoprotein cholesterol and hyperuricemia in diabetic patients
Source: Front Endocrinol (Lausanne). 2026 Mar 17;17:1790201. doi: 10.3389/fendo.2026.1790201 (PMC13035721; doi:10.3389/fendo.2026.1790201)
Supplement: Supplementary file 1 [file Table1.docx]

| **Table S1** Baseline characteristics of diabetic participants by hyperuricemia status (Chinese diabetic cohort). | | | | |
| --- | --- | --- | --- | --- |
|  | **Overall**  **(n=248)** | **Non-hyperuricemia**  **(n=205)** | **Hyperuricemia**  **(n=43)** | **P value** |
| **Age (years)** | 62.93 ± 11.46 | 63.18 ± 11.31 | 61.74 ± 12.22 | 0.458 |
| **Sex, n (%)** |  |  |  | 0.766 |
| **Female** | 116 (46.77%) | 95 (46.34%) | 21 (48.84%) |  |
| **Male** | 132 (53.23%) | 110 (53.66%) | 22 (51.16%) |  |
| **Smokers, n (%)** | 51 (20.56%) | 40 (19.51%) | 11 (25.58%) | 0.371 |
| **Alcohol use, n (%)** | 29 (11.69%) | 22 (10.73%) | 7 (16.28%) | 0.303 |
| **Hypertension, n (%)** | 171 (68.95%) | 137 (66.83%) | 34 (79.07%) | 0.115 |
| **CVDs, n (%)** | 42 (16.94%) | 35 (17.07%) | 7 (16.28%) | 0.900 |
| **BMI (kg/m^2^)** | 25.40 ± 3.58 | 25.20 ± 3.48 | 26.39 ± 3.90 | 0.047 |
| **Albumin (g/L)** | 40.71 ± 4.58 | 40.66 ± 4.80 | 40.96 ± 3.37 | 0.694 |
| **HbA1c (%)** | 9.65 ± 5.51 | 9.41 ± 2.18 | 10.79 ± 12.41 | 0.135 |
| **TG (mg/dL)** | 193.51 ± 247.65 | 170.50 ± 162.95 | 303.18 ± 465.64 | 0.001 |
| **TC (mg/dL)** | 178.09 ± 48.07 | 175.59 ± 45.57 | 189.99 ± 57.70 | 0.074 |
| **HDL-C (mg/dL)** | 50.90 ± 14.32 | 51.85 ± 14.53 | 46.38 ± 12.45 | 0.022 |
| **LDL-C (mg/dL)** | 95.85 ± 29.64 | 93.76 ± 28.83 | 105.83 ± 31.73 | 0.015 |
| **E-sdLDL-C (mg/dL)** | 35.64 ± 15.39 | 33.84 ± 13.14 | 44.23 ± 21.53 | <0.001 |
| **Scr (umol/L)** | 67.11 ± 23.64 | 64.73 ± 20.20 | 78.48 ± 33.85 | <0.001 |
| **SUA (μmol/L)** | 310.86 ± 86.44 | 284.00 ± 64.31 | 438.91 ± 59.54 | <0.001 |

| **Table S2** Regression analyses of E-sdLDL-C associations with hyperuricemia and SUA (Chinese diabetic cohort). | | | |
| --- | --- | --- | --- |
| **E-sdLDL-C** | **Model 1** | **Model 2** | **Model 3** |
| **Hyperuricemia** | **OR (95%CI) P value** | | |
| **Per SD increase** | 1.79 (1.30, 2.45) <0.001 | 1.90 (1.33, 2.71) <0.001 | 1.98 (1.35, 2.92) <0.001 |
| **Quantiles** |  |  |  |
| **Q1** | Reference | Reference | Reference |
| **Q2** | 1.19 (0.38, 3.76) 0.770 | 1.13 (0.35, 3.62) 0.8343 | 1.44 (0.42, 5.00) 0.5616 |
| **Q3** | 1.79 (0.61, 5.29) 0.289 | 1.81 (0.60, 5.45) 0.2944 | 1.83 (0.54, 6.20) 0.3343 |
| **Q4** | 4.44 (1.64, 12.03) 0.003 | 4.56 (1.60, 13.00) 0.0046 | 5.03 (1.56, 16.16) 0.007 |
| **P for tend** | 0.001 | 0.002 | 0.004 |
| **SUA levels** | **β (95%CI) P value** | | |
| **Per SD increase** | 23.51 (13.12, 33.91) <0.001 | 23.95 (12.96, 34.94) <0.001 | 23.44 (13.13, 33.75) <0.001 |
| **Quantiles** |  |  |  |
| **Q1** | Reference | Reference | Reference |
| **Q2** | -9.92 (-39.17, 19.34) 0.507 | -5.43 (-34.22, 23.36) 0.712 | 0.03 (-26.79, 26.85) 0.998 |
| **Q3** | 22.28 (-6.97, 51.53) 0.137 | 21.44 (-7.88, 50.77) 0.153 | 18.36 (-9.20, 45.93) 0.193 |
| **Q4** | 56.35 (27.10, 85.60) <0.001 | 54.28 (24.15, 84.42) <0.001 | 50.80 (22.13, 79.48) <0.001 |
| **P for tend** | <0.001 | <0.001 | <0.001 |
| OR: odds ratio.  95% CI: 95% confidence interval.  Model 1: non-adjusted.  Model 2: adjusted for age, sex, smoking, and alcohol use.  Model 3: adjusted for Model 2+hypertension, CVDs, BMI, albumin, Hba1c, and Scr. | | | |
